# Cognitive and Affective Theory of Mind across Adulthood

**DOI:** 10.3390/brainsci12070899

**Published:** 2022-07-09

**Authors:** Simona Raimo, Maria Cropano, María Dolores Roldán-Tapia, Lidia Ammendola, Daniela Malangone, Gabriella Santangelo

**Affiliations:** 1 Department of Medical and Surgical Sciences, “Magna Graecia” University of Catanzaro, 88100 Catanzaro, Italy; 2 Department of Psychology, University of Campania “Luigi Vanvitelli”, 81100 Caserta, Italy; maria.cropano@studenti.unicampania.it (M.C.); lidia.ammendola@studenti.unicampania.it (L.A.); daniela.malangone@studenti.unicampania.it (D.M.); gabriella.santangelo@unicampania.it (G.S.); 3 Department of Psychology, University of Almería, 04120 Almería, Spain; mdroldan@ual.es

**Keywords:** cognitive theory of mind, affective theory of mind, social cognition, aging, adulthood

## Abstract

Background: Theory of mind (ToM) is a fundamental aspect of social cognition. Previous studies on age-related changes in mentalizing processes have provided conflicting results. This study aims to investigate the age-related changes in the cognitive and affective components of ToM throughout adulthood. Methods: Two hundred and thirty-eight healthy participants divided into five age groups (18–40 years old; 41–50 years old; 51–60 years old; 61–70 years; 71–80 years old) underwent tasks assessing the cognitive (ToM Picture Sequencing Task, TMPS, and the Advanced Test of ToM, ATT) and affective (Reading the Mind in the Eyes Task, RMET, and the Emotion Attribution Task, EAT) components of ToM, in both verbal and nonverbal modality. Results: Regarding affective ToM, both the youngest- and middle-old adult groups (61 to 80 years) performed worse than the young and youngest-middle adult groups (18 to 50 years) in the RMET, but no significant differences were found in the EAT. Regarding cognitive ToM, the middle-old adult group (71 to 80 years) performed worse than the young adult group (18 to 40 years) only in the TMPS, but no significant differences were found in the ATT. Conclusion: Rather than a general decline in ToM, our results provide evidence regarding selective changes in ToM in older adults, further confirming the dissociation of cognitive and affective ToM.

## 1. Introduction

Theory of mind (ToM) is the ability to attribute mental states (i.e., intentions, emotions, desires, beliefs) to oneself and others, and to understand that others have beliefs, desires, and intentions different from one’s own [1]. It is a fundamental aspect of social cognition that influences interactions with others and individual behavior across a range of interpersonal contexts. Neuroimaging and behavioral studies [2,3,4,5] have demonstrated that it is a multidimensional construct that can be dissociable into two components. The first is the cognitive component which refers to a cognitive understanding of others’ knowledge, intentions, and beliefs, whereas the second is the affective component which refers to the processing of others’ emotions and feelings [3,6]. Most studies investigate these two ToM components during childhood, finding that the ability to understand another person’s beliefs and reactions develops gradually: first-order beliefs develop approximately around 4–5 years of age; second-order beliefs around 6–7 years of age; and third-order beliefs around 7 years of age [7,8,9], and through adolescence and young adulthood [10,11]. In the same way, the ability to infer other’s emotional states and feelings develops gradually: facial expression recognition and the role of desire in emotion develops approximately around 2–4 years of age [12]; the identification of external causes of emotion and the comprehension of the roles of beliefs and memory in emotion develop around 5–6 years of age [13]; the ability to distinguish between felt and expressed emotions develops around 6–7 years of age [14]; and an awareness of emotional regulation strategies develops around 8–9 years of age [14]. However, in the last two decades, there has been growing interest in investigating ToM abilities in healthy aging, but the results are still controversial. Indeed, some studies have shown that the cognitive component of ToM begins to decline from the age of 70 years [15,16], whereas other studies have shown that it declines only in old and very old age (from the age of 80 years, [17,18]), and other studies do not report aging-related changes at all [19,20]. Similarly, some studies have shown that the affective component of ToM declines in aging [21,22], whereas other studies have shown that it remains unchanged during aging (e.g., [23]). To date, a few studies have investigated healthy aging regarding both cognitive and affective ToM components using a within-subject design and yielding mixed findings. For instance, Duval and colleagues [24] demonstrated that older adults aged 70 years or more scored worse on cognitive and affective ToM tasks compared to young (from the age of 21 to 34 years) and middle-aged adults (from the age of 45 to 59 years), whereas Li and colleagues [25] and Wang and Su [26] found aging effects (over 70 years old) on the cognitive but not the affective ToM component. Additionally, other studies investigated the effect of healthy aging on both cognitive and affective ToM components using the same task. For instance, Fischer and colleagues [27] used the Yoni task [3] to assess both cognitive and affective ToM and found that older adults aged 64 to 87 years had poorer cognitive and affective ToM performance compared to young adults (from the age of 17 to 27 years), whereas Bottiroli and colleagues [28], assessing both cognitive and affective ToM using the Faux Pas Test [29], found that the young adult group (from the age of 19 to 27 years) outperformed the old adults (from the age of 60 to 82 years) on the cognitive component of ToM, but not on the affective one. Recently, Baksh and colleagues [30], investigating the effect of healthy aging on both cognitive and affective ToM using the Edinburgh Social Cognition Test, found that middle- (from the age of 45 to 60 years) and old-aged (from the age of 65 to 85 years) adults had significantly poorer performance on both ToM components compared to young adults (from the age of 18 to 35 years).

These differences reported in the studies referenced above are likely related to several methodological issues (i.e., task types used), and to the age band considered (e.g., young adults vs. older adults and middle-aged adults vs. older adults). For instance, the affective component of ToM is usually assessed by means of ToM tasks that mostly rely on visual decoding abilities (e.g., emotion attribution from faces; [31]) rather than by means of verbal tasks that require more reasoning abilities [26,28]. Additionally, some studies compare young adults with very broad middle- (e.g., from 40 to 59 years old [20]) and older-age bands (e.g., from 60 to 85 years old [32]), whereas others consider more restricted age band groups (e.g., from 50 to 90 years old spread across four decades [18]).

With similar divergence in the behavioral literature findings, the application of neuroimaging, useful for understanding how aging affects ToM processes, has also been utilized. Indeed, Castelli and colleagues [23] found that performance in a task of facial emotion recognition was associated with the activation of the right inferior frontal gyrus (IFG), a brain area associated with the visual memory encoding of faces [33], both in younger (from the age of 21 to 30 years) and older adults (from the age of 60 to 78 years), whereas the left IFG, a brain region typically associated with verbal memory [33] and selection between alternative answers [34], was significantly more activated in older adults than younger adults, suggesting that, in aging, impairments in domain-specific ToM performance are distinguished from general domain processing skills. Additionally, the results obtained by Moran and colleagues [35], who assessed the ability to spontaneously extract the intention information from moving shapes [36], showed that older adults (over 70 years old) had a reduced activation of the dorsomedial prefrontal cortex compared to young adults (over 23 years old), suggesting that older adults would be impaired at extracting intention information from moving shapes, regardless of the use of verbal materials, working memory, and executive selection demands.

To clarify these issues, the aim of the present study is to investigate possible age-related changes in cognitive and affective components of ToM by means of various types of ToM tasks (i.e., in verbal and nonverbal modalities) in a large sample of healthy adults, comparing ToM task performances in more specific and restricted age bands. We hypothesize that aging is associated with a reduction in cognitive and affective ToM performance, and that these age differences are affected by task modality (e.g., verbal, nonverbal).

## 2. Materials and Methods

### 2.1. Participants

Two hundred and thirty-eight healthy individuals participated in this study. They were grouped according to adulthood stage [28,37,38] into five age bands: the group of young adults consisted of 50 participants aged from 18 to 40 years old; the group of youngest-middle adults consisted of 50 participants aged from 41 to 50 years old; the group of middle adults consisted of 50 participants aged from 51 to 60 years old; the group of youngest-old adults consisted of 50 participants aged from 61 to 70 years old; and the group of middle-old adults consisted of 38 participants aged from 71 to 80 years old. Each age group was composed of 50 participants including 25 females and 25 males, but the middle-old adult group was composed of 18 females and 20 males. In order to detect an effect of η^2^ = 0.075 with 95% power in one-way between-subject ANOVA (analysis of variance; five groups, alpha = 0.05), G × Power suggested that we would need a total sample size of 235 participants. Participants were recruited through personal contacts and by word of mouth from the Psychology Department of the University of Campania “Luigi Vanvitelli” (Italy). All participants were native Italians, had no current mental health disorders such as depression or anxiety, according to the *Diagnostic and Statistical Manual of Mental Health Disorders, 5th Edition* (DSM-5; [39]), and obtained normal age- and education-adjusted scores in the Montreal Cognitive Assessment (MoCA; [40]), according to Italian normative data [41], and in Raven’s Coloured Progressive Matrices (RCPM; [42]), according to Italian normative data [43], excluding the presence of general cognitive impairment and deficits in abstract reasoning. The study was designed in accordance with the ethical standards laid down in the 1964 Declaration of Helsinki and was approved by the Ethical Committee of the University of Campania “Luigi Vanvitelli,” Caserta, Italy (protocol number 0001363, 14 January 2022). All participants signed an informed consent form to participate in the study and did not receive any payment for their participation. Descriptive statistics of the five age groups are reported in Table 1.

### 2.2. Neuropsychological Assessment

Global cognitive functioning was assessed using the MoCA [40,41] which consists of 12 subtasks exploring the following cognitive domains: i. memory, evaluated by means of the delayed recall of five words after two verbal presentations; ii. visuospatial abilities, evaluated by copying a cube and a clock-drawing task; iii. executive function, evaluated by means of a brief version of the Trail Making B task, a phonemic fluency task, and a verbal abstraction task; iv. attention, concentration, and working memory, evaluated by means of a sustained attention task, a serial subtraction task, and forward and backward digit span tasks; v. language, evaluated by a naming task, the repetition of two syntactically complex sentences and the phonemic fluency task; vi. temporal and spatial orientation, assessed by means of structured queries (i.e., “Tell me the year and month, tell me the name of this place”). The MoCA total scores ranged from 0 to 30, with higher scores indicating better performance.

Abstract reasoning was assessed using the RCPM [42,43], which is composed of three sets/scales (A, Ab, and B) with 12 items. Each item consists of a figure with a missing part which the participant needs to complete by choosing one of six alternative responses. There is only one correct answer for each item. The total scores ranged from 0 to 36, with higher scores indicating better performance.

### 2.3. ToM Assessment

#### 2.3.1. Affective Component of ToM

The affective component of ToM was evaluated for nonverbal modalities through the administration of the Reading the Mind in the Eyes Task (RMET; [44,45]) and for verbal modalities through the Emotion Attribution Task (EAT; [46]). The Italian version of RMET consisted of 36 greyscale photographs of male and female eyes depicting emotional states presented consecutively. Each photograph revealed a complex emotional or mental state, such as ‘thoughtful’ or ‘worried’. Participants were asked to choose the affective state that best described the eyes and the feeling of each individual, choosing between one of four possible emotions presented at each corner of the photograph (for example, item f in the RMET, see [44]). One point was assigned for each correct answer and the total scores ranged from 0 to 36, with higher scores indicating better performance. In the Italian version, the internal consistency (Cronbach’s alpha) was 0.605 [45].

In the modified Italian version of the EAT, participants were presented with 35 short stories describing emotional situations and were asked to report what the main protagonists felt in those situations. Five stories were designed to elicit attributions of sadness, five for fear, five for embarrassment, five for disgust, five for happiness, five for anger, and five for envy. One point was assigned for each correct answer and the total scores ranged from 0 to 35 with higher scores indicating better performance.

#### 2.3.2. Cognitive Component of ToM

The cognitive component of ToM was evaluated in nonverbal modalities through the administration of the Theory of Mind Picture Sequencing Task (TMPS; [47]) and in verbal modalities through the Advanced Test of ToM (ATT; [48]). The TMPS consisted of six cartoon picture stories of four cards each, depicting two scenarios where two characters cooperate, two scenarios where one character deceives a second character, and two scenarios where two characters cooperate in deceiving a third. The cards were presented covered and in a random order, and participants were asked to uncover and arrange them in a logical sequence of events (for example, item f in the TMPS, see [49]). For each story, two points were given if the first and fourth cards were correctly ordered, and one point if the second and the third card were correctly ordered, achieving a total score ranging from 0 to 36 with higher scores indicating better performance. Moreover, participants were asked to answer 23 questions to assess the comprehension of the characters’ mental states in the stories. The questions investigated the participants’ comprehension of first-, second-, and third-order beliefs, as well as their understanding of cheating and their comprehension of reciprocity, and then posed two real questions. One point was assigned for each correctly answered question. The scale demonstrated a good internal consistency (Cronbach’s alpha coefficient 0.86; [47]).

The Italian version of the ATT consisted of 13 stories which described several situations in which two or more characters interacted with each other in social contexts. Participants were asked to explain the reasons why the characters behaved as they did. The total scores ranged from 0 to 13 with higher scores indicating better performance.

### 2.4. Procedure

All participants underwent a neuropsychological battery, including the MoCA [40,41], the RCPM [42,43], and the tasks investigating ToM abilities. The entire evaluation was performed in the morning in a quiet experimental room at the Laboratory of Neuropsychology of the University of Campania “Luigi Vanvitelli” by a trained psychologist and completed in a single session that lasted approximately 1 h.

### 2.5. Statistical Analysis

To verify the normality of data distribution for the ToM tasks scores, we used the Kolmogorov–Smirnov test. It showed that most variables recorded (ToM task total scores) were not normally distributed (RMET: D(238) = 1.52, *p* = 0.020; EAT (D(238) =1.11, *p* = 0.164; TMPS D(238) = 2.23, *p* < 0.001; ATT D(238) = 2.36, *p* < 0.001), and for this reason non-parametric analyses were performed.

To evaluate the association between cognitive and affective ToM across the adult lifespan, correlations between four ToM task total scores (RMET, EAT, TMPS and ATT) were performed separately for the five age groups (young adult group, youngest-middle adult group, middle adult group, youngest-old adult group, and middle-old adult group) using Spearman’s rank correlation coefficient. The Spearman rank correlation coefficient values range from −1 (indicating an inverse linear association) through 0 (indicating no association at all) to +1 (indicating perfect positive linear association), with values of more than 0.70, 0.40–0.69, and less than 0.40 indicating, strong, moderate, and weak association, respectively.

To evaluate age-related differences in the ToM task performances, considering the role of sex and global cognitive functioning, we performed a rank analysis of covariance (ANCOVA; [50]) with the five age groups (young adult group, youngest-middle adult group, middle adult group, youngest-old adult group, and middle-old adult group) as the fixed factor, covarying for sex (male = 0, female = 1) and for global cognitive functioning (MoCA total score) as independent variables, and the scores in RMET, EAT, TMPS and ATT as dependent variables. In particular, to evaluate age-related differences in the ability to recognize and attribute specific emotions and different higher-order mental states, we performed further non-parametric analyses (i.e., Kruskal–Wallis or Mann–Whitney) on the EAT and TMPS subscores (i.e., EAT subscores: sadness, fear, embarrassment, happiness, disgust, anger and envy; TMPS subscores: first-, second-, and third-order beliefs, reality, reciprocity, deception, and cheating detection) only among those age groups that differed significantly in the EAT or TMPS total scores.

To evaluate if age-related differences in ToM abilities are domain-specific, we also performed a rank ANCOVA on the average of the standardized scores (calculated for each participant) for RMET and EAT to obtain the affective ToM component overall score, and on the average of the standardized scores for TMPS and ATT to obtain the cognitive ToM component overall scores.

**Figure 1:**
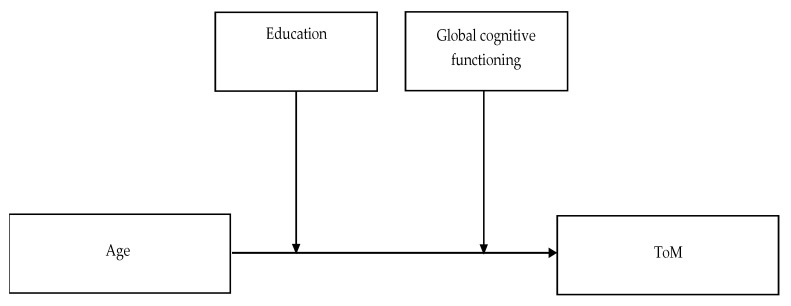
The moderation effect of education and global cognitive functioning on age’s direct effect on ToM abilities. Abbreviation: ToM—Theory of Mind.

Finally, moderation analyses were conducted using the bootstrapping technique to assess the moderating role of education and global cognitive functioning on the relation between aging and ToM abilities. The bootstrapping moderation analysis was performed using the PROCESS macro for SPSS [51]. Thus, to examine the strength of the relation between age and ToM abilities under different values of education and global cognitive functioning, the age was inputted as the independent variable, ToM tasks scores (RMET, EAT, TMPS and ATT scores) were inputted as the outcome variables, and education and global cognitive functioning were inputted as moderator variables (model 2, see Figure 1). Outliers above and below the two standard deviations were removed. The significance level was set at alpha level < 0.05, and Bonferroni correction for multiple comparisons was applied. All analyses were performed using SPSS v. 23.0 (SPSS, Inc. Chicago, IL, United States).

## 3. Results

### 3.1. Correlation Analyses of ToM Tasks among Age Groups

The correlation analyses performed separately on the five age groups showed no significant correlations (after Bonferroni’s correction) between the ToM task scores in the young adult group (see Table 2). Instead, significant correlations were found in the youngest-middle adult group and in the middle adult group between the EAT total score and the ATT total score (see Table 2); in the youngest-old adult group between the RMET total score and the TMPS score; and in the middle-old adult group between the RMET score and the ATT total score, and between the EAT and ATT total scores (see Table 2).

### 3.2. Comparison Analyses among Age Groups for ToM Tasks

**Figure 2:**
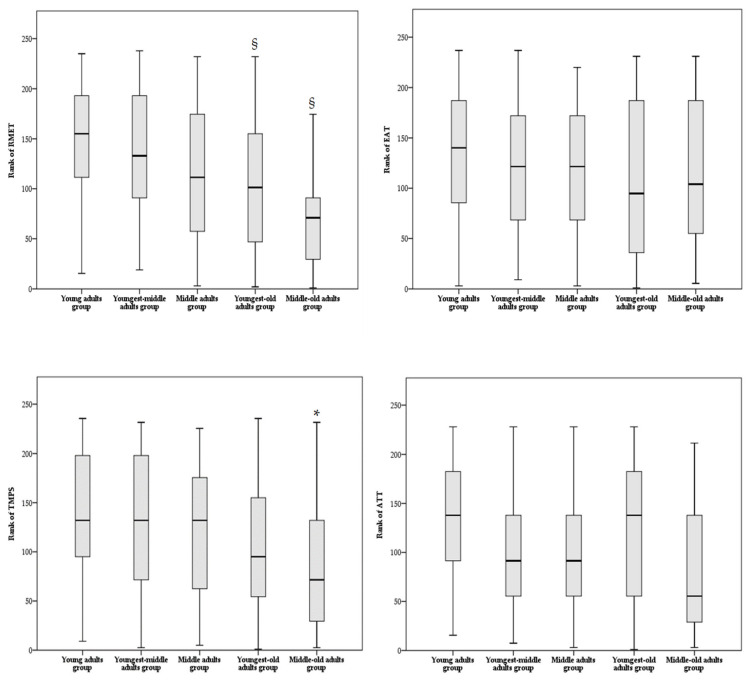
Box plot of comparison analyses among age groups on ToM tasks. Multiple comparisons between age groups were adjusted by Bonferroni correction. Abbreviations: RMET—Reading Mind in the Eyes Test; EAT—Emotion Attribution Task; TMPS—Theory of Mind Picture Sequencing Task; ATT—Advanced Test of ToM. * significantly different from young adult group, § significantly different from young and youngest-middle adult groups.

Regarding the affective component of ToM, the rank ANCOVA showed a significant main effect of age group for the nonverbal modality ToM task (RMET: F = 6.061, *p* < 0.001) but not for the verbal modality ToM task (EAT: F = 0.355, *p* = 0.840). Global cognitive functioning (F = 5.497, *p* = 0.020), but not sex (F = 0.141, *p* = 0.708), was a significant covariate for RMET total score, whereas no significant covariates (F ≤ 3.254, *p* ≥ 0.073) were found for EAT total score. Bonferroni-corrected post hoc comparisons showed that the youngest-old and the middle-old adult groups reported lower performances on RMET compared to the young and the youngest-middle adult groups (see Table 3 and Figure 2).

Regarding the cognitive component of ToM, the rank ANCOVA showed a significant main effect of age group for the nonverbal modality ToM task (TMPS: F = 2.911, *p* = 0.023) but not for the verbal modality ToM task (ATT: F = 2.091, *p* = 0.084). No significant covariates for TMPS or ATT total score were found (global cognitive functioning: F ≤ 1.668, *p* ≥ 0.198; sex: F ≤ 0.894, *p* ≥ 0.346). Bonferroni-corrected post hoc comparisons showed a significant difference between the young adult group and the middle-old adult group, with the latter reporting lower performances on TMPS (see Table 3). In particular, the middle-old adult group achieved significantly lower scores than the young adult group on TMPS subscores assessing reciprocity (Mann–Whitney U = 736, *p* = 0.032) and cheating detection (Mann–Whitney U = 808, *p* = 0.037); no other differences were found among the other TMPS subscores (Mann–Whitney U ≥ 848, *p* ≥ 0.360).

### 3.3. Comparison Analyses among Age Groups for ToM Affective and Cognitive Outcomes

The rank ANCOVA on affective ToM component overall score (average of standardized scores of RMET and EAT) showed a significant main effect of age group (F = 2.750, *p* = 0.030), although this result did not survive Bonferroni correction. The rank ANCOVA on the cognitive ToM component overall score (average of standardized scores of TMPS and ATT) showed a significant main effect of age group (F = 4.338, *p* = 0.002). No significant covariates were found (global cognitive functioning: F = 2.369, *p* = 0.126; sex: F = 0.411, *p* = 0.522). Bonferroni-corrected post hoc comparisons showed that the middle-old adult group reported lower performance for the cognitive ToM component overall score compared to the young and the youngest-middle adult groups.

### 3.4. Moderation Role of Education and Global Cognitive Functioning in Relation to Aging and ToM Abilities

Regarding the affective ToM component, the overall model was significant for the nonverbal modality ToM task (RMET: R^2^ = 0.275, F = 12.697, *p* < 0.001) with no significant interaction between age and education (b = 0.002, t = 1.871, *p* = 0.063) or between age and global cognitive functioning (b = 0.001, t = 0.799, *p* = 0.425). There was no significance for the verbal modality ToM task (EAT: R^2^ = 0.032, F = 1.103, *p* = 0.360).

**Figure 3:**
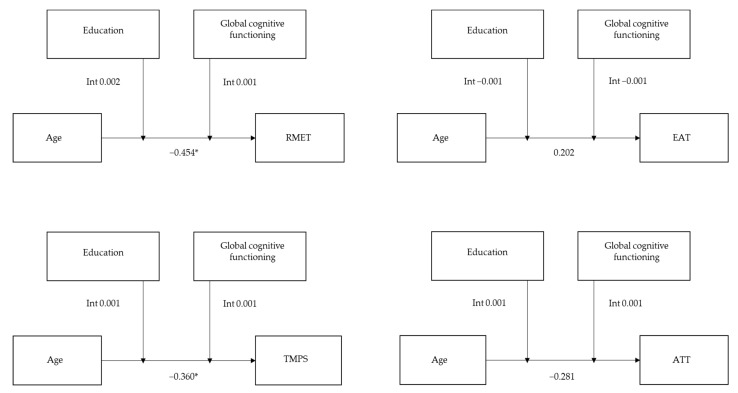
Moderating effect of education and global cognitive functioning on the association between age and each ToM task. Abbreviations: RMET—Reading Mind in the Eyes Test; EAT—Emotion Attribution Task; TMPS—Theory of Mind Picture Sequencing Task; ATT—Advanced Test of ToM. * *p* ≤ 0.05.

Regarding the cognitive ToM component, the overall model was significant for the nonverbal modality ToM task (TMPS: R^2^ = 0.134, F = 5.118, *p* < 0.001) with no significant interaction between age and education (b = 0.001, t = 1.179, *p* = 0.240) or between age and global cognitive functioning (b = 0.001, t = 0.749, *p* = 0.454). There was no significance for the verbal modality ToM task (ATT: R^2^ = 0.048, F = 1.637, *p* = 0.153; see Figure 3).

### 3.5. Summary of the Results

In this study, we found significant and positive correlations between affective and cognitive ToM in almost all age groups, except for the young adult group. Regarding ToM differences among age groups, we found that: the youngest-old and the middle-old adult groups reported lower RMET performances compared to the young and youngest-middle adult groups. Moreover, the middle-old adult group reported lower performances on the TMPS compared to the young adult group; in particular, the middle-old adult group achieved significantly lower scores than the young adult group in terms of the TMPS subscores assessing reciprocity and cheating detection. In addition, the middle-old adult group reported lower performance on the cognitive ToM component overall score compared to the young and the youngest-middle adult groups. Finally, we found that the relation between aging and ToM tasks for nonverbal modality was independent of education and global cognitive functioning level.

## 4. Discussion

The present study aimed to investigate the aging effect on both affective and cognitive ToM during the adult lifespan, considering the influence of the modality (verbal or nonverbal) of the tasks used to assess ToM abilities and controlling for global cognitive functioning and sex. The main findings of the present study were that: (i) the affective component of ToM declines earlier than the cognitive component during adulthood (from the age of 60 years old), and such worsening is specific to the ability to infer others’ emotions and decode emotional expressions (nonverbal modality), rather than the ability to reason on emotional mental states from social stories (verbal modality); (ii) the cognitive component of ToM worsens with advancing age (from the age of 70 years old), and such worsening is independent of the modality of the task used (i.e., verbal or nonverbal); (iii) age predicts ToM ability regardless of education and global cognitive functioning level.

These results suggest that ToM ability is impaired in aging, and this is not fully dependent on age-related difficulties, general cognition or fluid intelligence [16,52]. Indeed, Sullivan and Ruffman [16] and Keightley and colleagues [52] found that age-related ToM abilities in older adults aged over 70 years were independent of changes in fluid abilities (i.e., working memory, and executive function) and age-related cognitive decline, suggesting that general cognitive functioning and ToM could engage distinct, although overlapping, patterns of neural activation [20,53], as reported in studies on neuropathological decline [54,55].

Moreover, preserved ToM ability found in verbal modality tasks might result from greater knowledge about social relationships, and thus from an improved crystallized intelligence (for a systematic review, see [56]). In line with previous studies [31,57], our results demonstrate that, from the age of 60 years old, the ability to decode emotion from facial cues (eyes) worsens, suggesting that emotion recognition processing changes from young to old adult age, and would seem to be associated with different patterns of neural activation. Indeed, an fMRI study [23] revealed that young and older adults during the RMET showed a common activation of the right inferior frontal gyrus (IFG), a region associated with the visual memory encoding of faces (e.g., [33]) and a different activation in the left IFG and the anterior cingulate cortex. In particular, older adults also showed the increased activation of the left IFG, a region that is typically associated with verbal memory, probably to compensate for any difficulties they may have had in mentalizing ability, while the young adults showed the increased activation of the anterior cingulate cortex, a brain region specifically involved in inferring other’s mental states [58]. Thus, the cognitive ToM component seems to decline from 70 years onwards, according to previous studies that have explored the cognitive ToM component in healthy individuals [18,32,59]. This result confirms the effects of aging on the ability to infer the cognitive states, beliefs, thoughts, or intentions of other people, which may be due to age-related changes in executive control processes [15,60]. However, this issue remains controversial, as some studies find no relation between cognitive ToM and executive task performance in aging [24,61].

Moreover, the comparison of the subscores of the cognitive ToM tasks (i.e., TMPS) showed that the older adult group (71 to 80 years old) had selective difficulty in the detection of reciprocity and cheating. Thus, the decline in ToM could explain the social disengagement that usually occurs in aging, both as a reduction in prosocial behavior, such as sharing, comforting, and helping others [62], and as an impaired ability to recognize false claims and suspicious intentions to avoid potentially harmful social interactions, placing them at elevated risk to be tricked or scammed [63].

Finally, in accordance with previous behavioral and neuroimaging studies [3,64], our correlation analysis results provide additional insight into age-related differences in ToM abilities, showing that affective and cognitive components of ToM are independent for young adults, whereas they are strongly associated in older age.

Thus, starting from our findings, further studies should enroll samples of very old adults (from 80 years old) and also consider and better investigate the possible influence of neuropsychological and behavioral profiles (e.g., executive functioning, depression) on ToM functioning in aging.

We acknowledge some limitations of our study. First, the lack of standardization across studies and the scarcity of psychometric information in the tasks used to assess ToM abilities might have affected our findings [65]. Indeed, there is a long-standing debate in the literature about how ToM should be defined and measured. Especially, there is criticism of the RMET, which is based on the ability to recognize complex emotional states rather than reason with affective mental states [66]. Second, our study only focused on affective and cognitive components of ToM, although an increasing amount of evidence suggests the multifaced nature of ToM [67].

## 5. Conclusions

In conclusion, our results provide evidence regarding significant changes in both cognitive and affective ToM components in older adults that could partly explain a worsening of social functioning, health, and psychological well-being with aging [68]. Therefore, elderly people over the age of 61 should take social cognition measures by partaking in routine neuropsychological screening for the early identification of difficulties in intention and emotion recognition, and thus benefit from specific strategies to enhance socio-cognitive abilities (i.e., [69,70]).

## Figures and Tables

**Table 1:** Descriptive statistics of the five age groups.

	Young Adult Group (N = 50)		Youngest-Middle Adult Group (N = 50)		Middle Adult Group (N = 50)		Youngest-Old Adult Group (N = 50)		Middle-Old Adult Group (N = 38)	
	Mean ± SD	Range	Mean ± SD	Range	Mean ± SD	Range	Mean ± SD	Range	Mean ± SD	Range
Age	28.84 ± 6.11	18–40	46.10 ± 2.98	41–50	55.36 ± 2.55	51–60	64.44 ± 2.94	61–70	73.76 ± 2.36	71–80
Sex (F/M)	25/25		25/25		25/25		25/25		18/20	
Education °	14.72 ± 2.51	8–18	12.82 ± 3.81	8–18	11.86 ± 3.47	5–18	12.30 ± 4.14	5–18	9.57 ± 3.83	5–18
MoCA	27.56 ± 1.82	23–30	26.41 ± 2.12	20–30	25.58 ± 2.24	21–30	23.48 ± 3.69	14–29	22.07 ± 3.24	12–27
RCPM	34.26 ± 2.30	26–36	31.67 ± 3.68	18–36	30.94 ± 3.97	21–36	28.87 ± 5.17	14–36	26.76 ± 6.64	13–36
MoCA *	25.15 ± 1.88	20.59–28.85	25.07 ± 2.02	21.15–28.65	25.47 ± 2.28	21.51–30	24.03 ± 2.81	16.11–27.98	24.11 ± 3.21	16.40–27.72
RCPM *	29.34 ± 2.43	21.20–35.90	28.64 ± 3.24	17.30–34.30	29.62 ± 3.68	19.30–36	29.20 ± 3.55	17.90–35.90	28.57 ± 5.83	12.10–36

Abbreviations: N—number of participants; MoCA—Montreal Cognitive Assessment; RCPM—Raven’s Coloured Progressive Matrices; F— female; M—male; SD—standard deviation; °—years of study considering the highest academic degree achieved; *—age- and education-adjusted scores. Adjusted scores of Raven’s Coloured Progressive Matrices and MoCA are not statistically different among groups (RCPM: H = 2.511, *p* = 0.643; MoCA: H = 5.249, *p* = 0.263).

**Table 2:** Correlations between ToM task total scores for each age group.

**Young Adult Group**					
		RMET	EAT	TMPS	ATT
RMET	r_rho_	1	0.145	0.285	0.236
	p		0.316	0.047	0.114
EAT	r_rho_	-	1	0.372	0.327
	p			0.008	0.027
TMPS	r_rho_	-	-	1	0.308
	p				0.039
ATT	r_rho_	-	-	-	1
	p				
**Youngest-Middle Adult Group**					
		RMET	EAT	TMPS	ATT
RMET	r_rho_	1	0.318	0.267	0.329
	p		0.025	0.061	0.019
EAT	r_rho_	-	1	0.372	0.464 *
	p			0.008	0.001
TMPS	r_rho_	-	-	1	0.134
	p				0.352
ATT	r_rho_	-	-	-	1
	p				
**Middle Adult Group**					
		RMET	EAT	TMPS	ATT
RMET	r_rho_	1	0.156	0.293	0.032
	p		0.278	0.039	0.824
EAT	r_rho_	-	1	0.334	0.453 *
	p			0.018	0.001
TMPS	r_rho_	-	-	1	0.320
	p				0.024
ATT	r_rho_	-	-	-	1
	p				
**Youngest-Old Adult Group**					
		RMET	EAT	TMPS	ATT
RMET	r_rho_	1	−0.082	0.524 *	0.192
	p		0.571	<0.001	0.181
EAT	r_rho_	-	1	0.197	0.362
	p			0.169	0.010
TMPS	r_rho_	-	-	1	0.046
	p				0.752
ATT	r_rho_	-	-	-	1
	p				
**Middle-Old Adult Group**					
		RMET	EAT	TMPS	ATT
RMET	r_rho_	1	0.429	0.293	0.489 *
	p		0.007	0.078	0.003
EAT	r_rho_	-	1	0.213	0.580 *
	p			0.206	<0.001
TMPS	r_rho_	-	-	1	0.141
	p				0.426
ATT	r_rho_	-	-	-	1
	p				

Abbreviations: RMET—Reading Mind in the Eyes Test; EAT—Emotion Attribution Task; TMPS—Theory of Mind Picture Sequencing Task; ATT—Advanced Test of ToM. * *p* ≤ 0.003, after Bonferroni correction.

**Table 3:** ToM task performance for the five age groups.

	Young Adult Group	Youngest-Middle Adult Group	Middle Adult Group	Youngest-Old Adult Group	Middle-Old Adult Group	Age Group		
	Mean ± SD	Mean ± SD	Mean ± SD	Mean ± SD	Mean ± SD	F	*p*	η^2^
RMET	26.74 ± 4.07	26.34 ± 3.76	24.56 ± 4.68	23.82 ± 5.42 *§	22 ± 5.29 *§	6.061	<0.001	0.127
EAT	26.44 ± 4.82	25.50 ± 4.73	25.62 ± 4.30	24.34 ± 6.23	25.47 ± 4.88	0.355	0.841	0.008
TMPS	51.91 ± 4.21	50.62 ± 5.64	49.62 ± 6.05	49.14 ± 5.73	47.48 ± 6.05 *	2.911	0.023	0.066
ATT	10.19 ± 1.62	9.22 ± 1.90	9.24 ± 1.93	9.34 ± 2.17	8.62 ± 2.01	2.091	0.084	0.049

Abbreviations: SD— standard deviation; RMET—Reading Mind in the Eyes Test; EAT—Emotion Attribution Task; TMPS—Theory of Mind Picture Sequencing Task; ATT—Advanced Test of ToM. * significantly different from young adult group; § significantly different from youngest-middle adult group.

## Data Availability

Data are available on request.

## References

[1] Premack D., Woodruff G. (1978). Does the chimpanzee have a theory of mind?. Behav. Brain Sci..

[2] Shamay-Tsoory S.G., Tomer R., Goldsher D., Berger B.D., AharonPeretz J. (2004). Impairment in cognitive and affective empathy in patients with brain lesions: Anatomical and cognitive correlates. J. Clin. Exp. Neuropsychol..

[3] Shamay-Tsoory S.G., Aharon-Peretz J. (2007). Dissociable prefrontal networks for cognitive and affective theory of mind: A lesion study. Neuropsychologia.

[4] Hynes C.A., Baird A.A., Grafton S.T. (2006). Differential role of the orbital frontal lobe in emotional versus cognitive perspective-taking. Neuropsychologia.

[5] Völlm B.A., Taylor A.N., Richardson P., Corcoran R., Stirling J., McKie S., Deakin J.F., Elliott R. (2006). Neuronal correlates of theory of mind and empathy: A functional magnetic resonance imaging study in a nonverbal task. NeuroImage.

[6] O’Brien M., Miner Weaver J.M., Nelson J.A., Calkins S.D., Leerkes E.M., Marcovitch S. (2011). Longitudinal association between children‘s understanding of emotion and theory of mind. Cogn. Emot..

[7] Wimmer H., Perner J. (1983). Beliefs about beliefs: Representation and constraining function of wrong beliefs in young children’s understanding of deception. Cognition.

[8] Perner J., Wimmer H. (1985). “John Thinks That Mary Thinks That…” attribution of second-order beliefs by 5- to 10- year-old children. J. Exp. Child Psychol..

[9] Astington J.W., Dack L.A., Haith M.M., Benson J.B. (2008). Theory of Mind. Encyclopedia of Infant and Early Childhood Development.

[10] Vetter N.C., Altgassen M., Phillips L., Mahy C.E.V., Kliegel M. (2013). Development of affective theory of mind across adolescence: Disentangling the role of executive functions. Dev. Neuropsychol..

[11] Gabriel E.T., Oberger R., Schmoeger M., Deckert M., Vockh S., Auff E., Willinger U. (2021). Cognitive and affective Theory of Mind in adolescence: Developmental aspects and associated neuropsychological variables. Psychol. Res..

[12] MacDonald P.M., Kirkpatrick S.W., Sullivan L.A. (1996). Production of facial expressions of emotion in preschool children. Percept. Mot. Skills.

[13] Ruffman T., Keenan T.R. (1996). The belief-based emotion of suprise: The case for a lag in understanding relative to false belief. Dev. Psychol..

[14] Pons F., Harris P.L., de Rosnay M. (2004). Emotion comprehension between 3 and 11 years: Developmental periods and hierarchical organization. Eur. J. Dev. Psychol..

[15] Phillips L.H., Bull R., Allen R., Insch P., Burr K., Ogg W. (2011). Lifespan aging and belief reasoning: Influences of executive function and social cue decoding. Cognition.

[16] Sullivan S., Ruffman T. (2004). Social understanding: How does it fare with advancing years?. Br. J. Psychol..

[17] Maylor E.A., Moulson J.M., Muncer A., Taylor L.A. (2002). Does performance on theory of mind tasks decline in old age?. Br. J. Psychol..

[18] Charlton R.A., Barrick T.R., Markus H.S., Morris R.G. (2009). Theory of mind associations with other cognitive functions and brain imaging in normal aging. Psychol. Aging.

[19] Saltzman J., Strauss E., Hunter M., Archibald S. (2000). Theory of mind and executive functions in normal human aging and Parkinson’s disease. J. Int. Neuropsychol. Soc..

[20] MacPherson S.E., Phillips L.H., Della Sala S. (2002). Age, executive function, and social decision making: A dorsolateral prefrontal theory of cognitive aging. Psychol. Aging.

[21] Mahy C.E.V., Vetter N., Kühn-Popp N., Löcher C., Krautschuk S., Kliegel M. (2014). The influence of inhibitory processes on affective theory of mind in young and old adults. Neuropsychol. Dev. Cogn..

[22] Grainger S.A., Henry J.D., Phillips L.H., Vanman E.J., Allen R. (2017). Age deficits in facial affect recognition: The influence of dynamic cues. J. Gerontol. B Psychol. Sci. Soc. Sci..

[23] Castelli I., Baglio F., Blasi V., Alberoni M., Falini A., Liverta-Sempio O., Nemni R., Marchetti A. (2010). Effects of aging on mindreading ability through the eyes: An fMRI study. Neuropsychologia.

[24] Duval C., Piolino P., Bejanin A., Eustache F., Desgranges B. (2011). Age effects on different components of theory of mind. Conscious. Cogn..

[25] Li X., Wang K., Wang F., Tao Q., Xie Y., Cheng Q. (2013). Aging of theory of mind: The influence of educational level and cognitive processing. Int. J. Psychol..

[26] Wang Z., Su Y. (2013). Age-related differences in the performance of theory of mind in older adults: A dissociation of cognitive and affective components. Psychol. Aging.

[27] Fischer A.L., O’Rourke N., Loken Thornton W. (2017). Age Differences in Cognitive and Affective Theory of Mind: Concurrent Contributions of Neurocognitive Performance, Sex, and Pulse Pressure. J. Gerontol. B. Psychol. Sci. Soc. Sci..

[28] Bottiroli S., Cavallini E., Ceccato I., Vecchi T., Lecce S. (2016). Theory of Mind in aging: Comparing cognitive and affective components in the faux pas test. Arch. Gerontol. Geriatr..

[29] Stone V.E., Baron-Cohen S., Knight R.T. (1998). Frontal lobe contributions to theory of mind. J. Cogn. Neurosci..

[30] Baksh R.A., Abrahams S., Auyeung B., MacPherson S.E. (2018). The Edinburgh Social Cognition Test (ESCoT): Examining the effects of age on a new measure of theory of mind and social norm understanding. PLoS ONE.

[31] Slessor G., Phillips L.H., Bull R. (2007). Exploring the specificity of age-related differences in theory of mind tasks. Psychol. Aging.

[32] Bernstein D.M., Thornton W.L., Sommerville J.A. (2011). Theory of mind through the ages: Older and middle-aged adults exhibit more errors than do younger adults on a continuous false belief task. Exp. Aging Res..

[33] Kelley W.M., Miezin F.M., McDermott K.B., Buckner R.L., Raichle M.E., Cohen N.J., Ollinger J.M., Akbudak E., Conturo T.E., Snyder A.Z. (1998). Hemispheric specialization in human dorsal frontal cortex and medial temporal lobe for verbal and nonverbal memory encoding. Neuron.

[34] Thompson-Schill S.L., D’Esposito M., Aguirre G.K., Farah M.J. (1997). Role of left inferior prefrontal cortex in retrieval of semantic knowledge: A reevaluation. Proc. Natl. Acad. Sci. USA.

[35] Moran J.M., Jolly E., Mitchell J.P. (2012). Social-cognitive deficits in normal aging. J. Neurosci..

[36] Castelli F., Happé F., Frith U., Frith C. (2000). Movement and mind: A functional imaging study of perception and interpretation of complex intentional movement patterns. Neuroimage.

[37] Raimo S., Di Vita A., Boccia M., Iona T., Cropano M., Gaita M., Guariglia C., Grossi D., Palermo L. (2021). The Body across the Lifespan: On the Relation between Interoceptive Sensibility and High-Order Body Representations. Brain Sci..

[38] Lee S.B., Oh J.H., Park J.H., Choi S.P., Wee J.H. (2018). Differences in youngest-old, middle-old, and oldest-old patients who visit the emergency department. Clin. Exp. Emerg. Med..

[39] American Psychiatric Association (2013). Diagnostic and Statistical Manual of Mental Disorders.

[40] Nasreddine Z.S., Phillips N.A., Bédirian V., Charbonneau S., Whitehead V., Collin I., Cummings J.L., Chertkow H. (2005). The Montreal Cognitive Assessment, MoCA: A brief screening tool for mild cognitive impairment. J. Am. Geriatr. Soc..

[41] Santangelo G., Siciliano M., Pedone R., Vitale C., Falco F., Bisogno R., Siano P., Barone P., Grossi D., Santangelo F. (2015). Normative data for the Montreal Cognitive Assessment in an Italian population sample. Neurol. Sci..

[42] Raven J.C. (1947). Progressive Matrices 1947.

[43] Spinnler H., Tognoni G. (1987). Standardizzazione e taratura italiana di test neuropsicologici. Ital. J. Neurol. Sci..

[44] Baron-Cohen S., Jolliffe T., Mortimore C., Robertson M. (1997). Another advanced test of theory of mind: Evidence from very high functioning adults with autism or asperger syndrome. J. Child. Psychol. Psychiatry.

[45] Vellante M., Baron-Cohen S., Melis M., Marrone M., Petretto D.R., Masala C., Preti A. (2013). The “Reading the Mind in the Eyes” test: Systematic review of psychometric properties and a validation study in Italy. Cogn. Neuropsychiatry.

[46] Blair R.J., Cipolotti L. (2000). Impaired social response reversal. A case of ‘acquired sociopathy’. Brain.

[47] Brüne M. (2003). Theory of mind and the role of IQ in chronic disorganized schizophrenia. Schizophr. Res..

[48] Prior M., Marchi S., Sartori G. (2003). Social Cognition and Behavior. A Tool for Assessment. Cognizione Sociale e Comportamento. Uno Strumento per la Misurazione, ED.

[49] Lissek S., Peters S., Fuchs N., Witthaus H., Nicolas V., Tegenthoff M., Juckel G., Brüne M. (2008). Cooperation and deception recruit different subsets of the theory-of-mind network. PLoS ONE.

[50] Quade D. (1967). Rank analysis of covariance. J. Am. Stat. Assoc..

[51] Hayes A.F. (2013). Introduction to Mediation, Moderation, and Conditional Process Analysis: A Regression-Based Approach.

[52] Keightley M.L., Winocur G., Burianova H., Hongwanishkul D., Grady C.L. (2006). Age effects on social cognition: Faces tell a different story. Psychol. Aging.

[53] Schurz M., Radua J., Aichhorn M., Richlan F., Perner J. (2014). Fractionating theory of mind: A meta-analysis of functional brain imaging studies. Neurosci. Biobehav. Rev..

[54] Poletti M., Enrici I., Adenzato M. (2012). Cognitive and affective Theory of Mind in neurodegenerative diseases: Neuropsychological, neuroanatomical and neurochemical levels. Neurosci. Biobehav. Rev..

[55] Belfort T., Simões J.P., Santos R.L., Lacerda I., Dourado M.C.N. (2020). Social cognition: Patterns of impairments in mild and moderate Alzheimer’s disease. Int. J. Geriatr. Psychiatry.

[56] Moran J.M. (2013). Lifespan development: The effects of typical aging on theory of mind. Behav. Brain Res..

[57] Kynast J., Quinque E.M., Polyakova M., Luck T., Riedel-Heller S.G., Baron-Cohen S., Hinz A., Witte A.V., Sacher J., Villringer A. (2020). Mindreading From the Eyes Declines With Aging—Evidence from 1603 Subjects. Front. Aging Neurosci..

[58] Amodio D.M., Frith C.D. (2006). Meeting of minds: The medial frontal cortex and social cognition. Nature reviews. Neuroscience.

[59] German T.P., Hehman J.A. (2006). Representational and executive selection resources in ‘theory of mind’: Evidence from compromised belief-desire reasoning in old age. Cognition.

[60] Bailey P.E., Henry J.D. (2008). Growing less empathic with age: Disinhibition of the self-perspective. J. Gerontol. B Psychol. Sci. Soc. Sci..

[61] Cavallini E., Lecce S., Bottiroli S., Palladino P., Pagnin A. (2013). Beyond false belief: Theory of mind in young, young-old, and old-old adults. Int. J. Aging Hum. Dev..

[62] Obisesan T.O., Gillum R.F. (2009). Cognitive function, social integration and mortality in a U.S. national cohort study of older adults. BMC Geriatr..

[63] James B.D., Boyle P.A., Bennett D.A. (2014). Correlates of susceptibility to scams in older adults without dementia. J. Elder. Abuse. Negl..

[64] Kalbe E., Schlegel M., Sack A.T., Nowak D.A., Dafotakis M., Bangard C., Brand M., Shamay-Tsoory S., Onur O.A., Kessler J. (2010). Dissociating cognitive from affective theory of mind: A TMS study. Cortex.

[65] Quesque F., Rossetti Y. (2002). What do theory-of-mind tasks actually measure? Theory and practice. Perspect. Psychol. Sci..

[66] Altschuler M.R., Trevisan D.A., Wolf J.M., Naples A.J., Foss-Feig J.H., Srihari V.H., McPartland J.C. (2021). Face perception predicts affective theory of mind in autism spectrum disorder but not schizophrenia or typical development. J. Abnorm. Psychol..

[67] Schaafsma S.M., Pfaff D.W., Spunt R.P., Adolphs R. (2015). Deconstructing and reconstructing theory of mind. Trends Cogn. Sci..

[68] Hall J.A., Andrzejewski S.A., Yopchick J.E. (2009). Psychosocial correlates of interpersonal sensitivity: A meta-analysis. J. Nonverbal Behav..

[69] Cavallini E., Ceccato I., Bertoglio S., Francescani A., Vigato F., Ianes A.B., Lecce S. (2021). Can theory of mind of healthy older adults living in a nursing home be improved? A randomized controlled trial. Aging Clin. Exp. Res..

[70] Lecce S., Bottiroli S., Bianco F., Rosi A., Cavallini E. (2015). Training older adults on Theory of Mind (ToM): Transfer on metamemory. Arch. Gerontol. Geriatr..

